# The dark side of the court: **masculinity**, machiavellianism, and perfectionism in sport and e-sport contexts

**DOI:** 10.3389/fpsyg.2026.1799445

**Published:** 2026-05-07

**Authors:** Judit Glavanits

**Affiliations:** Department of Modern Technology and Cybersecurity Law, Faculty of Law and Political Sciences, Széchenyi István University, Györ, Hungary

**Keywords:** dark personality traits, e-sport psychology, maladaptive perfectionism, masculine norms, performance-oriented environments, sport psychology

## Abstract

**Introduction:**

Sport and e-sport environments function as performance-oriented spaces where masculine norms emphasizing dominance and success are salient. While these norms influence psychological outcomes, the mechanisms linking them to maladaptive self-regulation require further clarification. This study investigated the associations between masculine norm endorsement, Machiavellianism, and perfectionism, specifically testing the mediating role of Machiavellian traits.

**Methods:**

A cross-sectional survey was conducted with 297 adult (65,9% male, Mage ≈ 24,4 years) participants, including athletes, e-sport players, and non-athletes. Measures included the Multicultural Masculinity Ideology Scale (MMIS), the Machiavellianism Personality Scale (MPS), and the Short Almost Perfect Scale (SAPS). Data were analyzed using Welch's ANOVA, correlation, and mediation models with 5,000 bootstrap resamples.

**Results:**

Masculine norm endorsement correlated positively with Machiavellianism but showed no direct association with perfectionism. Machiavellianism was positively related to maladaptive perfectionism, and the observed pattern of associations was consistent with an indirect pathway between masculine norm endorsement and maladaptive perfectionism via Machiavellianism (indirect effect: *p* = 0.013), although no significant total effect was found. No significant indirect effect was observed for adaptive perfectionism.

**Discussion:**

Machiavellianism appears to account for the observed association pattern linking masculinity norms to maladaptive perfectionistic self-regulation. In competitive “bastions of masculinity,” these traits may prioritize strategic success over ethical conduct, highlighting the need for psychological screening to balance high performance with athlete well-being.

## 1 Introduction

Sport and e-sport contexts are widely recognized as performance-oriented social environments in which gendered norms, particularly those associated with masculinity, are highly salient. Competitive settings emphasize dominance, emotional control, success orientation, and resilience under pressure—traits that closely align with culturally dominant masculine ideals (Connell, 1987; Connell and Messerschmidt, 2005; Messner and Sabo, 1994; Soares et al., 2022; Raggiotto and Scarpi, 2023). Within such environments, masculinity is not merely an individual identity characteristic, but a socially regulated performance shaped by institutional expectations, peer dynamics, and evaluative pressures (Courtenay, 2000; Vandello and Bosson, 2013). Sport therefore represents a critical context for examining how masculine norms are endorsed, enacted, and potentially translated into psychological functioning. Voorhees (2014) introduced the concept of neoliberal masculinity to describe how competitive gaming (e-sport) cultures promote self-discipline, individual responsibility, constant self-optimization, and emotional detachment as normative masculine ideals. Within e-sport contexts, success is framed as the outcome of personal investment, skill maximization, and relentless performance monitoring, while structural conditions and collective responsibility are downplayed. This form of masculinity aligns closely with market-oriented logics, positioning players as entrepreneurial subjects who must continuously regulate themselves to remain competitive. Importantly, such environments valorize instrumental attitudes toward others and toward the self, creating a cultural context in which performance, control, and strategic self-regulation are central to masculine identity construction.

Importantly, endorsement of masculine norms does not necessarily imply deep internalization or stable identity integration. Contemporary gender theories emphasize the performative and precarious nature of masculinity, suggesting that masculine status must be repeatedly demonstrated and is particularly sensitive to threat in competitive, male-dominated settings (Pleck, 1995; Vandello and Bosson, 2013; Raveendran, 2023). In such contexts, individuals may strategically align with masculine norms to maintain legitimacy, status, or group belonging, even in the absence of strong personal identification. Empirical research in sport psychology supports this distinction, showing that normative endorsement of masculine ideals can coexist with heterogeneous patterns of internalization and psychological outcomes (Steinfeldt et al., 2012; Dawson and Hammer, 2020; Tan et al., 2022).

This strategic dimension of norm endorsement becomes especially relevant when considered alongside dark personality traits, particularly Machiavellianism. Machiavellianism is characterized by manipulativeness, instrumental social behavior, emotional detachment, and a flexible approach to moral rules in the pursuit of personal goals (Christie and Geis, 1970; Paulhus and Williams, 2002; Jones and Paulhus, 2014). Individuals high in Machiavellianism tend to treat social norms as tools rather than intrinsic moral guides, adopting or discarding them based on situational utility. Competitive sport environments, with their strong emphasis on outcomes, ranking, and performance evaluation, may be especially conducive to such instrumental orientations (Kavussanu, 2019; Nicholls et al., 2017). While narcissism has traditionally been more directly linked to overt masculinity, dominance, and self-enhancement in sport contexts, it primarily reflects grandiosity and the need for admiration rather than the strategic use of norms. In contrast, Machiavellianism is uniquely positioned to explain how masculine norms are selectively enacted, internalized, or disengaged as instruments of performance-oriented self-regulation, making it particularly relevant for understanding when normative endorsement translates into maladaptive psychological functioning.

Empirical research further suggests that Machiavellian traits in sport are not merely associated with isolated unethical acts but reflect a broader instrumental orientation toward competition, interpersonal relations, and rule systems. Athletes high in Machiavellianism may be more likely to approach sport as a context in which achieving advantage justifies the use of flexible and instrumental strategies, including those that extend beyond strict adherence to fair play norms (Christie and Geis, 1970; Paulhus and Williams, 2002). While competitive sport is inherently structured as a zero-sum context, the distinguishing feature of Machiavellian tendencies lies in the increased likelihood of employing manipulative, strategically exploitative, or ethically ambiguous behaviors (e.g., tactical fouling, psychological provocation) to secure success.

Several studies indicate that Machiavellianism is positively associated with moral disengagement mechanisms in sport, such as the rationalization of rule violations, minimization of harm, and diffusion of responsibility within team settings (Kavussanu, 2019; Nicholls et al., 2017; Greitemeyer, 2022). These cognitive strategies allow athletes to maintain a positive self-image while engaging in behaviors that conflict with formal ethical standards. Importantly, such mechanisms are often embedded in everyday sport practices and coaching cultures, where tactical fouling, psychological provocation, or deliberate exploitation of loopholes are framed as signs of competitive intelligence rather than moral compromise. In this sense, Machiavellianism may be less a deviant trait in sport contexts than a socially reinforced adaptation to competitive demands.

Empirical evidence also suggests that Machiavellian traits are differentially expressed across sport types and competitive levels. Research comparing individual and team sports has found higher Machiavellian orientations among athletes in team-based and highly competitive environments, where social dynamics, hierarchy, and strategic coordination play a central role (Dhormare, 2016). Similarly, studies on elite and semi-elite athletes indicate that higher performance pressure and status insecurity are associated with stronger endorsement of instrumental and manipulative strategies (González-Hernández et al., 2020). These findings align with theoretical accounts emphasizing that Machiavellian tendencies are activated under conditions of high stakes, evaluation, and perceived threat to status. The relevance of Machiavellianism becomes particularly salient when considering its intersection with gender norms in sport. Masculine ideals emphasizing toughness, dominance, emotional restraint, and winning at all costs may create a normative climate in which Machiavellian strategies are not only tolerated but implicitly rewarded. Within such climates, behaviors associated with manipulation, control, and moral flexibility can be reframed as expressions of competitive masculinity rather than ethical violations (Connell and Messerschmidt, 2005; Messner and Sabo, 1994). This reframing is especially evident in narratives that valorize “mental toughness” and “competitive edge,” often at the expense of empathy and fairness. Importantly, Machiavellianism has also been linked to broader psychological outcomes in sport beyond overt behavior. Research indicates that athletes high in Machiavellian traits show elevated levels of control orientation, conditional self-worth, and performance-contingent self-evaluation, which may increase vulnerability to maladaptive self-regulatory patterns (Nicholls et al., 2017; Bryan et al., 2023). These characteristics suggest that Machiavellianism functions not only as an interpersonal strategy but also as an internal regulatory framework through which athletes interpret success, failure, and personal value (Nogueira et al., 2019). Such internalization may have downstream consequences for wellbeing, particularly in contexts where performance is unstable or publicly evaluated. Taken together, these findings indicate that Machiavellianism occupies a central position in the psychological ecology of competitive sport. Rather than operating as an isolated dark trait, it appears to reflect a broader alignment between performance-oriented norms, masculine ideals, and instrumental approaches to both others and the self. This perspective underscores the importance of examining Machiavellian traits as a potential mechanism through which masculine norm endorsement becomes psychologically consequential in sport and e-sport contexts.

Perfectionism represents another central psychological construct in sport and e-sport contexts, where performance is continuously monitored, compared, and evaluated. Contemporary models distinguish between adaptive forms of perfectionism, characterized by high personal standards and goal striving, and maladaptive forms, marked by excessive self-criticism, fear of failure, and rigid performance expectations (Hewitt and Flett, 1991; Stoeber and Otto, 2006). While adaptive perfectionism has been associated with persistence and motivation, maladaptive perfectionism consistently predicts negative outcomes such as anxiety, burnout, and impaired wellbeing in athletes (Hill et al., 2018; Reinhardt et al., 2019). Maladaptive perfectionism shares important conceptual features with Machiavellian functioning, including heightened control orientation, conditional self-worth, and an instrumental view of performance outcomes as indicators of status and value. In contrast, direct links between masculine norm endorsement and perfectionism have produced mixed findings, suggesting that masculinity-related norms may not translate into maladaptive self-regulation in a straightforward manner (Dawson and Hammer, 2020; Hill et al., 2018). This raises the possibility that the psychological consequences of masculine norm endorsement depend on intermediary personality mechanisms that shape how norms are enacted and internalized. Despite growing interest in masculinity, dark personality traits, and perfectionism in sport psychology, empirical research has rarely examined these constructs within an integrated framework. Little is known about whether Machiavellian traits function as a psychological mechanism linking masculine norm endorsement to maladaptive perfectionism in sport and e-sport contexts. Addressing this gap is especially relevant given increasing concerns about athlete mental health, ethical performance climates, and the sustainability of high-performance cultures (Kavussanu, 2019; Denison et al., 2021).

The present study aimed to examine the associations between masculine norm endorsement, Machiavellian personality traits, and perfectionism among sport and e-sport participants. Based on the theoretical framework outlined above, the following hypotheses were formulated. H1: Masculine norm endorsement is positively associated with Machiavellian traits. H2: Machiavellianism is positively associated with maladaptive perfectionism. H3: Sport and e-sport participation are associated with distinct perfectionism profiles. H4: Machiavellianism mediates the relationship between masculine norm endorsement and maladaptive perfectionism. By integrating perspectives from gender theory, personality psychology, and sport psychology, this study seeks to clarify how socially endorsed norms become psychologically consequential in performance-oriented environments.

## 2 Materials and methods

### 2.1 Participants

The final sample consisted of 297 adult participants after data cleaning procedures. Two responses were excluded due to invariant responding across all questionnaire items, and one participant was excluded for being under 18 years of age. Participants were recruited through direct outreach to sport and e-sport organizations, social media platforms, and university mailing lists. The sample included individuals who reported regular participation in physical sports, e-sport activities, as well as non-participants.

The gender distribution of the sample was unbalanced, with 195 male (65.9%) and 101 female (34.1%), (one participant identified as “other” gender) participants. This distribution was not considered a sampling limitation but rather a deliberate methodological choice aligned with the study's theoretical focus. Prior research indicates that masculine norm endorsement and its psychological consequences are more strongly expressed and more variable among men, making male-focused sampling particularly informative for research on masculinity-related mechanisms (Connell and Messerschmidt, 2005; Pleck, 1995; Steinfeldt et al., 2012). This approach is consistent with previous research examining masculinity norms in performance-oriented environments, where theoretically driven oversampling of men is commonly employed to capture variance in masculinity-related constructs (Courtenay, 2000; Vandello and Bosson, 2013). Male and female participants did not differ substantially in age distribution. Participants' mean age was approximately 24.4 years (*SD* ≈ 8.3), with ages ranging from 18 to 64 years. Participants self-reported their involvement in sport and e-sport activities, including whether they engaged in physical sports or e-sport competitively or recreationally. These variables were used for group comparisons and exploratory analyses but were not treated as primary predictors in the mediation models. We did not explicitly defined e-sport activity in the questionnaire, participants were categorized as e-sport players if they reported regular engagement in e-sports, incorporated in the questionnaire as “regular, game-like activity on computer or console devices”. Regarding activity type, 200 participants reported engagement in physical sports, while 120 participants reported engagement in e-sports. A total of 82 participants indicated involvement in both physical sport and e-sport activities. Among e-sport participants, *n* = 18 reported competitive participation. Subgroups were not mutually exclusive, as participants could report engagement in both physical sport and e-sport activities. Participants were not asked to specify the exact type of sport or e-sport activity. This was a deliberate methodological choice, as the primary aim of the study was to examine general performance-oriented environments (i.e., physical sport vs. e-sport) rather than sport-specific effects. Accordingly, the analyses focused on the presence of engagement in these activity domains, which were considered theoretically more relevant to the constructs under investigation than the specific discipline itself. However, this limitation is acknowledged in the Discussion as a direction for future research.

## 2 Measures

### 2.2.1 Masculine norm endorsement and internalized masculinity

Masculine norm endorsement was assessed using the Multicultural Masculinity Ideology Scale—MMIS (Doss and Hopkins, 1998; Kozma et al., 2019), which measures agreement with culturally embedded masculine norms independently of self-reported behavior. The ideological subscale of the MMIS consists of 27 items rated on a five-point Likert scale ranging from 1 (*strongly disagree*) to 5 (*strongly agree*). Higher scores indicate stronger endorsement of masculine norms. In the present sample, the ideological subscale demonstrated good internal consistency (Cronbach's α = 0.79). Internalized masculinity was measured using the behavioral component of the MMIS, which assesses the extent to which individuals perceive their own behavior as conforming to masculine norms. This subscale contains 23 items rated on the same five-point Likert scale. Consistent with previous validation studies on Hungarian sample (Kozma et al., 2019), internal consistency was lower but acceptable for exploratory research (Cronbach's α = 0.61).

### 2.2.2 Machiavellian personality traits

Machiavellian traits were measured using the Machiavellianism Personality Scale (MPS; Dahling et al., 2009), adapted and validated in Hungarian by Talmácsi et al. (2012). The Hungarian short version consists of 15 items rated on a five-point Likert scale. The scale captures four dimensions: amoral manipulation, desire for control, distrust of others, and perceived control. Higher scores indicate stronger Machiavellian tendencies. In the present sample, the overall scale demonstrated acceptable internal consistency (Cronbach's α = 0.65), which is consistent with previous findings in mixed-gender and non-clinical and Hungarian samples (Talmácsi et al., 2012), also similar with Serbian findings (Grabovac and Dinić, 2022).

### 2.2.3 Perfectionism

Perfectionism was assessed using the Short Almost Perfect Scale (SAPS) (Rice et al., 2014) validated on Hungarian sample by Reinhardt et al. (2019), which distinguishes between adaptive and maladaptive dimensions of perfectionism. The scale consists of eight items rated on a seven-point Likert scale ranging from 1 (*strongly disagree*) to 7 (*strongly agree*). The Standards subscale reflects adaptive perfectionism, while the Discrepancy subscale captures maladaptive perfectionism characterized by perceived failure to meet high standards. In the present study, the SAPS demonstrated excellent internal consistency (Cronbach's α = 0.88). Exploratory factor analysis confirmed the expected two-factor structure, supporting the use of separate adaptive and maladaptive perfectionism scores in subsequent analyses.

### 2.3 Procedure and statistical analysis

Data analyses were conducted using jamovi (version 2.7), an open-source statistical software environment built on the R programming language. An *a priori* power analysis was conducted using G^*^Power 3.1 (Faul et al., 2009) for a linear multiple regression model with two predictors. The analysis indicated that a minimum sample size of *N* = 199 would be required to detect a small-to-moderate effect size (*f*^2^ = 0.05) with 80% power at α = 0.05. The final sample (*N* = 297) therefore exceeded this threshold, indicating adequate statistical power for the primary regression-based analyses. The selected effect size reflects a conservative estimate consistent with prior research in personality and sport psychology, where associations between psychological constructs are typically small to moderate. Descriptive statistics and reliability analyses (Cronbach's alpha) were computed for all measures. Preliminary analyses indicated deviations from normality for several variables (particularly Machiavellianism and maladaptive perfectionism), as assessed by skewness, kurtosis, and Shapiro–Wilk tests. Therefore, Spearman correlations were used where appropriate, while Pearson correlations were applied for variables meeting parametric assumptions. Group differences were tested using Welch's analysis of variance (ANOVA) to account for unequal variances and group sizes, as indicated by variance heterogeneity. To examine the hypothesized mediating role of Machiavellian traits (H4), mediation analyses were conducted using bootstrapped confidence intervals with 5,000 resamples. In these models, masculine norm endorsement was entered as the predictor variable, Machiavellianism as the mediator, and adaptive and maladaptive perfectionism as outcome variables. Indirect effects were considered statistically significant if the 95% confidence interval did not include zero. Given the cross-sectional design, mediation models are interpreted as tests of statistical associations rather than causal processes. Indirect effects may emerge even in the absence of a significant total effect, and alternative causal structures cannot be ruled out. Therefore, these analyses are interpreted cautiously and complemented by correlation and regression results. All statistical tests were two-tailed, with the significance level set at *p* < 0.05.

### 2.4 Ethics statement

The study was conducted in accordance with the ethical standards of the Declaration of Helsinki. Ethical approval was obtained from the Research Ethics Committee of the Széchenyi István University (approval number: SZE/ETT-58/2025). All participants provided informed consent prior to participation and were informed about the voluntary nature of the study, data confidentiality, and their right to withdraw at any time without penalty.

## 3 Results

### 3.1 Preliminary analysis

Descriptive statistics and reliability analyses indicated acceptable to good internal consistency for all measures. The ideological component of the Multicultural Masculinity Ideology Scale demonstrated good reliability (Cronbach's α = 0.79), while the behavioral/internalized masculinity component showed lower but acceptable reliability for exploratory research (Cronbach's α = 0.61). The Machiavellianism Personality Scale showed acceptable internal consistency (Cronbach's α = 0.65), and the Short Almost Perfect Scale demonstrated excellent reliability (Cronbach's α = 0.88).

Table 1 presents the Spearman rank-order correlations among the main study variables. Masculine norm endorsement showed a moderate positive association with internalized masculinity and a weaker but significant association with Machiavellian traits, while no significant relationship was observed with perfectionism. In contrast, Machiavellianism was positively associated with perfectionism as well as with both dimensions of masculinity. This pattern indicates that masculine norm endorsement is not directly related to perfectionism but is systematically linked to perfectionistic tendencies through Machiavellian personality traits.

**Table 1:** Spearman correlations among main study variables.

		Masculine ideology	Internalized masculinty	Perfectionism
Internalized masculinty	Spearman's rho	0.352	—	
	*p*-value	<0.001	—	
Perfectionism	Spearman's rho	0.011	0.062	—
	*p*-value	0.857	0.290	—
Machiavellian personality	Spearman's rho	0.161	0.351	0.214
	*p*-value	0.005	<0.001	<0.001

These correlation patterns provide the baseline structure of associations among the main variables. In particular, the absence of a direct association between masculine norm endorsement and perfectionism, combined with the significant association between Machiavellianism and perfectionism, suggests that masculine norm endorsement is not directly related to perfectionism but may be indirectly associated with perfectionistic tendencies via Machiavellian personality traits. Accordingly, subsequent analyses examine whether Machiavellian traits account for this pattern of associations.

### 3.2 Hypothesizes

#### 3.2.1 Masculine norms and internalized masculinity of athletes and e-sports players

To test Hypothesis 3, we first examined whether engagement in sport and e-sport contexts is associated with masculine norm endorsement. Group comparisons supported the higher level of masculine norm endorsement among sportsmen. Participants engaged in physical sport, e-sport, and competitive forms of both activities reported significantly higher masculine norm endorsement scores than non-participants. Welch's ANOVA revealed a significant main effect of sport participation on masculine norm endorsement, [*F*_(1.220)_ = 4.81, *p* = 0.029], with a similar effect observed for competitive physical sport participation, [*F*_(181.9)_ = 5.06, *p* = 0.027. Comparable patterns emerged for e-sport and competitive e-sport participation (all *p*s <0.05), indicating that engagement in performance-oriented sport contexts was associated with stronger endorsement of masculine norms. In contrast, no consistent group differences were observed for internalized masculinity among physical sport participants, [*F*_(1.180)_ = 1.11, *p* = 0.293]. E-sport participants showed significantly higher internalized masculinity scores compared to non-e-sport participants, [*F*_(1.248)_ = 9.63, *p* = 0.002]; however, this effect was not robust when analyses were restricted to male participants only.

These findings indicate that performance-oriented environments are associated with stronger endorsement of masculine norms, supporting the assumption that such contexts reinforce masculinity-related beliefs rather than uniformly shaping internalized identity.

#### 3.2.2 Machiavellian personality traits

Correlational analyses supported the hypothesis assuming masculine norm endorsement is positively associated with Machiavellian personality traits among sport and e-sport participants. Masculine norm endorsement showed a significant positive association with Machiavellianism (Pearson's *r* = 0.19, *p* < 0.001). This association remained significant, though slightly weaker, when analyses were conducted on the male subsample only (*r* = 0.15, *p* < 0.05). To further examine this relationship, a linear regression model was estimated with Machiavellianism as the outcome variable and masculine norm endorsement as the predictor, controlling for sport vs. e-sport participation. The overall model was significant and accounted for approximately 22% of the variance in Machiavellianism (*R*^2^ = 0.22). Masculine norm endorsement emerged as a significant predictor, whereas sport type did not contribute significantly to the model. Taken together, both correlation and regression results indicate that masculine norm endorsement is a significant predictor of Machiavellian tendencies, supporting Hypothesis 1.

#### 3.2.3 Perfectionism profiles of athletes and e-sport players

To test Hypothesis 3 (sport and e-sport participants exhibit different perfectionism profiles compared to non-athletes), perfectionism was examined as a multidimensional construct, distinguishing between adaptive (Standards) and maladaptive (Discrepancy) dimensions. Prior to group comparisons, an exploratory factor analysis was conducted on the Short Almost Perfect Scale to confirm its factorial structure within the present sample. The Bartlett's test of sphericity was significant, χ^2^ (28) = 1,370, *p* < 0.001, and the Kaiser–Meyer–Olkin measure indicated good sampling adequacy (KMO = 0.85). Using minimum residual extraction with oblimin rotation, a two-factor solution emerged, accounting for 64.7% of the total variance. This structure was consistent with the theoretical distinction between adaptive and maladaptive perfectionism and justified the use of separate subscale scores in subsequent analyses.

A two-way multivariate analysis of variance (MANOVA) was performed with sport participation (athlete vs. non-athlete) and gender (male vs. female) as independent variables, and adaptive and maladaptive perfectionism as dependent variables. Results indicated a significant multivariate main effect of sport participation, Wilks' λ = 0.961, [*F*_(2.291)_ = 5.89, *p* = 0.003], suggesting that athletes and non-athletes differed in their overall perfectionism profiles. However, univariate follow-up analyses revealed a more nuanced pattern. Adaptive perfectionism showed a weak, non-significant positive association with physical sport participation (*r* = 0.097, *p* = 0.097). Although athletes tended to report slightly higher adaptive perfectionism, this difference did not reach statistical significance. In contrast, e-sport participation was significantly and negatively associated with adaptive perfectionism (*r* = −0.231, *p* < 0.001), indicating that e-sport players reported lower levels of adaptive, standards-oriented perfectionism compared to non-e-sport participants. Maladaptive perfectionism was not significantly associated with either physical sport participation (*r* = −0.028, *p* = 0.637) or e-sport participation (*r* = −0.019, *p* = 0.747). These findings indicate that participation in sport or e-sport in itself was not linked to elevated maladaptive perfectionistic tendencies. Overall, Hypothesis 3. received only limited support, as no association was found for maladaptive perfectionism.

Gender showed a significant multivariate main effect on perfectionism (Pillai's Trace = 0.045, [*F*_(2.290)_ = 6.80, *p* = 0.001)]. Univariate analyses revealed significant gender differences for both adaptive perfectionism, [*F*_(1.291)_ = 13.46, *p* < 0.001], and maladaptive perfectionism, [*F*_(1.291)_ = 5.24, *p* = 0.023]. In line with previous findings, female participants reported higher levels of maladaptive perfectionism on average.

Overall, these findings suggest that participation in sport or e-sport contexts is not directly associated with maladaptive perfectionism, indicating that perfectionistic tendencies may be more strongly linked to personality characteristics than to activity type itself. Taken together, these results shown in subchapters 3.2.1–3.2.3 indicate that while sport and e-sport participation are associated with masculine norm endorsement, they are not directly linked to maladaptive perfectionism. In contrast, Machiavellianism shows consistent associations with both masculinity and perfectionism, suggesting a potentially central role in linking these constructs. These findings support Hypothesis 2. In addition to these analyses, mediation models were examined to assess whether the observed pattern of associations is consistent with an indirect pathway, beyond the direct and unique effects identified in the regression models.

#### 3.2.4 Mediation analysis: machiavellianism as a mediator between masculine norms and maladaptive perfectionism

Based on the observed correlation and regression patterns, mediation analysis was conducted to explore whether the association between masculine norm endorsement and maladaptive perfectionism is consistent with an indirect pathway via Machiavellianism. Hypothesis 4 proposed that the relationship between masculine norm endorsement and maladaptive perfectionism would be mediated by Machiavellian personality traits. A mediation analysis was conducted using bootstrapped confidence intervals (5,000 resamples), with masculine norm endorsement as the predictor, Machiavellianism as the mediator, and maladaptive perfectionism as the outcome variable. Results revealed a significant indirect effect of masculine norm endorsement on maladaptive perfectionism through Machiavellianism [*B* = 0.0309, SE = 0.0124, *Z* = 2.48, *p* = 0.013, 95% CI (0.0098, 0.0598)], with the confidence interval not including zero. In contrast, the direct effect of masculine norm endorsement on maladaptive perfectionism was not significant (*B* = −0.0183, SE = 0.0327, *p* = 0.576), nor was the total effect (*B* = 0.0126, SE = 0.0345, *p* = 0.714).

This pattern is consistent with an indirect-only mediation model; however, it does not provide strong evidence for a causal mechanism. Instead, the findings suggest that the relationship between masculine norm endorsement and maladaptive perfectionism may be accounted for by their shared association with Machiavellian traits. This interpretation is consistent with the regression results, which showed that Machiavellianism accounted for unique variance in maladaptive perfectionism, whereas masculine norm endorsement did not. Given the cross-sectional design, the direction of effects cannot be established, and alternative causal pathways remain plausible. Therefore, the mediation results should be interpreted as indicative of a potential pattern of associations rather than a definitive psychological process. Hypothesis 4. was partially supported, as a significant indirect effect was observed in the absence of a total effect.

A parallel mediation model using adaptive perfectionism as the outcome variable did not yield significant results. Neither the indirect effect through Machiavellianism (*B* = 0.009, *p* = 0.120) nor the direct effect (*p* = 0.158) reached statistical significance, and the total effect was only marginal (*p* = 0.073). These findings suggest that the observed association pattern is specific to maladaptive, rather than adaptive forms of perfectionism.

## 4 Discussion

The present study examined how masculine norm endorsement, Machiavellian personality traits, and perfectionism are interrelated in sport and e-sport contexts, with a particular focus on the psychological mechanisms through which socially endorsed masculine norms become psychologically consequential. By integrating perspectives from gender theory, personality psychology, and sport psychology, the findings both support and refine several influential theoretical assumptions in the existing literature.

### 4.1 Masculine norm endorsement does not directly predict maladaptive perfectionism

From the perspective of masculinity stress and gender role strain theories, the absence of a direct association between masculine norm endorsement and maladaptive perfectionism is particularly noteworthy. Classical masculinity stress models posit that endorsement of masculine norms is inherently linked to heightened vulnerability to stress, self-evaluative pressure, and maladaptive regulation when masculine status is perceived as threatened (Pleck, 1995; Courtenay, 2000). Under this framework, stronger endorsement of masculine norms would be expected to correspond directly with more rigid, self-critical performance regulation. The present findings challenge this simplified assumption. Masculine norm endorsement alone was not associated with either adaptive or maladaptive perfectionism, suggesting that endorsement of masculine norms does not automatically translate into perfectionistic self-pressure. Instead, these results align with more recent conceptualizations of masculinity as a precarious and context-dependent social performance rather than a stable internal stressor (Vandello and Bosson, 2013). From this perspective, masculine norms constitute a cultural resource that may or may not become psychologically burdensome, depending on how they are interpreted, enacted, and integrated at the individual level. Importantly, the present study extends masculinity stress models by identifying Machiavellianism as a key psychological mechanism that conditions when masculine norm endorsement becomes maladaptive. Rather than generating stress directly, masculine norms appear to contribute to maladaptive perfectionism primarily when filtered through an instrumental, control-oriented personality orientation that emphasizes performance, dominance, and strategic self-regulation. This refinement suggests that masculinity-related stress is not an inevitable consequence of norm endorsement but emerges selectively through specific personality-mediated pathways.

### 4.2 Machiavellianism as a mediating mechanism: support for instrumental norm-use theories

The present findings suggest that Machiavellian traits play a central role in the association between masculine norm endorsement and maladaptive perfectionism. While no direct relationship was observed between masculinity and maladaptive perfectionism, Machiavellianism showed consistent associations with both constructs, and the mediation analysis indicated a significant indirect effect. Consistent with prior research, Machiavellianism was positively associated with masculine norm endorsement (Christie and Geis, 1970; Paulhus and Williams, 2002) and maladaptive perfectionism (Nicholls et al., 2017; Bryan et al., 2023). These findings align with theoretical accounts suggesting that Machiavellian individuals tend to engage with social norms in an instrumental manner, using them strategically rather than internalizing them as moral imperatives (Jones and Paulhus, 2014). Within competitive sport and e-sport contexts—where dominance, success, and emotional control are emphasized—masculine norms may thus function as culturally legitimate frameworks that reinforce rigid self-regulation and performance-contingent self-worth. In this sense, the present results are consistent with the notion that dark personality traits may contribute to the maladaptive internalization of performance-related norms (Kavussanu, 2019; Nicholls et al., 2017). However, this pattern should be interpreted with caution. The absence of a significant total effect, together with the cross-sectional design, does not support a strong causal interpretation. Rather than reflecting a clear directional mechanism, the observed pattern may indicate that these constructs are linked through a shared dispositional tendency. Accordingly, the mediation results may reflect shared variance between constructs rather than a specific psychological pathway. Future research using longitudinal designs or latent variable approaches would be needed to disentangle these possibilities and to more clearly establish the direction of effects.

An important alternative interpretation of the present findings concerns the role of the Dark Factor of Personality (Moshagen et al., 2020). Recent research in personality psychology suggests that dark traits such as Machiavellianism are not fully independent constructs but rather represent specific manifestations of a broader underlying dispositional core reflecting antagonistic, self-serving tendencies (Hilbig et al., 2021). From this perspective, the observed association between Machiavellianism and maladaptive perfectionism may not necessarily reflect a directional psychological mechanism but could partly arise from shared variance attributable to this common core. Indeed, prior studies have shown that a substantial proportion of variance in dark traits is accounted for by their shared underlying factor, rather than their unique components. Accordingly, the mediation pattern identified in the present study should be interpreted with caution. It is possible that Machiavellianism does not function as a distinct mediating mechanism *per se* but rather captures the expression of a broader dispositional tendency that is also related to maladaptive perfectionism. This interpretation aligns with recent findings indicating that associations between dark traits and psychological outcomes often reflect shared variance rather than specific causal pathways.

### 4.3 Internalized masculinity vs. ideological endorsement: refining masculinity theory

A central contribution of the present study lies in its differentiation between internalized masculinity and masculine ideological endorsement, which are frequently conflated in empirical research. Our findings are consistent with masculinity stress models suggesting that self-critical performance regulation may function as a compensatory mechanism for maintaining masculine identity under perceived threat (Pleck, 1995; Vandello and Bosson, 2013). At high levels of Machiavellianism, however, this association disappeared entirely, indicating that masculine identity is no longer contingent on perfectionistic self-evaluation.

In contrast, when masculine ideology served as the outcome variable, maladaptive perfectionism was negatively associated with ideological endorsement at high levels of Machiavellianism. This finding challenges assumptions that perfectionistic pressure necessarily reinforces adherence to masculine norms. Instead, it suggests that individuals high in Machiavellian traits may distance themselves ideologically from masculine norms precisely when internal performance pressure increases, supporting the interpretation of masculinity as an instrumental rather than internalized construct among such individuals.

### 4.4 Implications for sport and e-sport psychology

Beyond their theoretical relevance, the present findings have important practical implications for sport psychologists and coaches working in performance-oriented environments. In line with previous research linking Machiavellian traits to instrumental goal pursuit, moral disengagement, and outcome-focused regulation in sport (Kavussanu, 2019; Nicholls et al., 2017), the results suggest that maladaptive perfectionism should not be treated as a uniform risk factor requiring identical intervention strategies. The absence of a direct association between masculine norm endorsement and maladaptive perfectionism indicates that endorsement of performance-oriented masculine values alone is insufficient for identifying athletes at risk of harmful self-regulatory patterns. Instead, the findings highlight the importance of considering underlying personality orientations—particularly Machiavellianism—when interpreting perfectionistic tendencies. From an applied perspective, this implies that psychological screening and assessment practices may benefit from jointly evaluating perfectionism and Machiavellian traits to differentiate between athletes whose maladaptive perfectionism reflects internalized self-critical pressure and those for whom rigid self-regulation serves a more instrumental, strategic function. This distinction has direct implications for intervention: athletes low in Machiavellianism may benefit most from approaches targeting self-compassion, flexible goal adjustment, and the decoupling of self-worth from performance outcomes (Hill et al., 2018), whereas athletes high in Machiavellian traits may respond more effectively to interventions framed around long-term performance sustainability, ethical boundaries, and the strategic costs of rigid control rather than emotional vulnerability *per se*. At the coaching level, the results further suggest that highly masculine, outcome-focused performance climates do not uniformly produce maladaptive outcomes but interact with individual personality profiles in shaping self-regulation. Accordingly, coaches and support staff may reduce the risk of maladaptive perfectionism by adopting differentiated feedback and motivational strategies that acknowledge these individual differences, rather than if increased pressure or norm reinforcement will have the same psychological effect across athletes. Taken together, these findings underscore the value of a personality-informed, context-sensitive approach to preventing and managing maladaptive perfectionism in sport and e-sport settings.

### 4.5 Limitations

Several limitations of the present study should be acknowledged, which also point toward promising directions for future research. First, the cross-sectional design precludes any causal conclusions regarding the relationships among masculine norm endorsement, Machiavellianism, and perfectionism. Although the mediation model was theoretically grounded and consistent with the observed pattern of associations, the temporal ordering of these constructs cannot be definitively established. Given the cross-sectional design, the results do not allow for causal conclusions. This limitation is common in personality and sport psychology research, where cross-sectional approaches are frequently used to examine complex psychosocial relationships. Second, all variables were assessed using self-report measures, which may introduce shared method variance and social desirability biases. This concern is particularly relevant when studying socially sensitive constructs such as masculinity, dark personality traits, and perfectionism. However, it should be noted that the use of self-report instruments remains the dominant methodological approach in this research domain, including studies on masculinity ideology (Steinfeldt et al., 2012), Machiavellianism in sport (Kavussanu, 2019), and perfectionism among athletes (Hill et al., 2018). Future research could strengthen the robustness of these findings by incorporating multi-method approaches, such as peer reports, behavioral indicators, or experimental manipulations of performance pressure. An additional limitation concerns the relatively low number of participants involved in e-sport and competitive e-sport activities. Although the overall sample size was adequate for the primary analyses, the subsamples of e-sport players (*n* = 121) and especially competitive e-sport players (*n* = 18) were comparatively small. This limited statistical power may have reduced the sensitivity of group-level comparisons and interaction effects involving e-sport participation, particularly when analyses were further stratified by gender or competitive status. Fourth, although the sample included participants from sport, e-sport, and non-sport backgrounds, the study did not differentiate systematically between specific sport types, competitive levels, or cultural sport environments. Prior research suggests that Machiavellian traits and masculinity-related processes may vary across individual vs. team sports, contact vs. non-contact disciplines, and elite vs. recreational contexts (Dhormare, 2016; González-Hernández et al., 2020). Future studies could build on the present findings by examining whether the identified mediating mechanisms are amplified or attenuated in specific performance ecologies. Finally, the present findings should be interpreted within the cultural context of the sample (Hungarian participants). Masculine norms, their ideological content, and their psychological consequences are shaped by broader sociocultural frameworks. While the mechanisms identified here are theoretically consistent with international literature, cross-cultural replication is necessary to determine their generalizability.

## 5 Conclusions

The present study examined the associations between masculine norm endorsement, Machiavellian personality traits, and perfectionism in sport and e-sport contexts. The findings indicate that masculine norm endorsement is not directly related to maladaptive perfectionism, but is consistently associated with Machiavellian traits, which in turn show a robust relationship with maladaptive perfectionism. This pattern of results is consistent with an indirect association, suggesting that the psychological consequences of masculine norm endorsement may depend on underlying personality mechanisms. These findings contribute to the literature by highlighting the importance of distinguishing between normative endorsement and personality-driven regulation processes in performance-oriented environments. Rather than functioning as a direct risk factor, masculine norms appear to become psychologically consequential primarily when filtered through instrumental, control-oriented personality traits.

Future research should build on these findings using longitudinal and latent-variable approaches to disentangle shared variance and clarify the direction of effects. Additionally, examining these mechanisms across different sport types and cultural contexts may further refine our understanding of how performance environments shape psychological functioning. Importantly, masculinity should be considered a historically and culturally embedded construct that evolves over time. As such, existing measurement instruments may not fully capture contemporary expressions of masculinity in rapidly changing performance environments such as e-sports. Future studies may therefore benefit from developing and applying updated masculinity measures that are more closely aligned with current socio-cultural dynamics. Furthermore, future research should more explicitly integrate recent advances in dark personality research, particularly approaches emphasizing the shared core of dark traits (e.g., the Dark Factor of Personality). Incorporating these perspectives may help to disentangle trait-specific effects from shared dispositional variance and provide a more precise understanding of the psychological mechanisms underlying performance-related self-regulation.

From a practical perspective, the present findings highlight the importance of adopting a differentiated approach in sport psychology and coaching practice. The results suggest that maladaptive perfectionism should not be treated as a uniform phenomenon but rather understood in the context of underlying personality characteristics, particularly Machiavellian tendencies. In applied settings, this implies that psychological support and mental training interventions may benefit from integrating personality-informed assessments to distinguish between athletes whose rigid self-regulation reflects internalized self-critical pressure and those for whom it serves a more instrumental, performance-oriented function. For coaches and sport psychologists, these findings underscore the need to tailor feedback, motivational strategies, and intervention approaches accordingly. While athletes lower in Machiavellian traits may benefit from interventions targeting self-compassion, flexibility, and the decoupling of self-worth from performance outcomes, those higher in Machiavellianism may respond more effectively to approaches emphasizing long-term performance sustainability, ethical boundaries, and the strategic costs of rigid control. Overall, fostering performance environments that balance achievement orientation with psychological well-being may help mitigate the risk of maladaptive self-regulation in both sport and e-sport contexts.

## Data Availability

The raw data supporting the conclusions of this article will be made available by the authors, without undue reservation.
